# The combination of three advanced endoscopic techniques achieves recanalization of anastomotic stenosis after pancreatogastrostomy

**DOI:** 10.1055/a-2587-9217

**Published:** 2025-05-22

**Authors:** Shan-Shan Hu, Jie Hou, Rui Huang, Wei-Hui Liu

**Affiliations:** 1 89669 Department of Gastroenterology and Hepatology, Sichuan Academy of Medical Sciences and Sichuan Provincial Peopleʼs Hospital, School of Medicine, University of Electronic Science and Technology of China, Chengdu, China

With the rise in cases of pancreaticogastrostomy, managing anastomotic stenosis has become particularly challenging. The most prevalent method, endoscopic ultrasound-guided pancreatic duct drainage (EUS-PD) surgery 1 2 3 , currently faces two main issues: one is the limited success rate of the procedure, and the other is the difficulty in achieving the widest possible drainage of pancreatic juice 4 . Here, we present a case of an anastomotic stricture following pancreaticogastrostomy, successfully treated by recanalizing the original stricture using a combination of three advanced endoscopic techniques, offering a potential approach for managing similar cases ( Video 1 ).

The combination of three advanced endoscopic techniques achieves recanalization of anastomotic stenosis after pancreatogastrostomy.Video 1

A male patient underwent laparoscopic duodenum-preserving pancreatic head resection with pancreaticogastrostomy for a pancreatic head hamartoma. Recently, he experienced recurrent pancreatitis due to an anastomotic stricture. Gastroscopy confirmed complete anastomotic occlusion, and the procedure was planned to reopen the occlusion and restore normal pancreatic duct function.

The stomach cavity was filled with water, and endoscopic ultrasound (EUS) was used to locate the pancreatic duct. The weakest part of the pancreatic-gastric anastomosis was identified, and cauterization marking was performed under EUS guidance to designate the starting point for the later dissection process using the endoscopic submucosal dissection (ESD) technique ( Fig. 1 ). A gastroscope with a transparent cap was then used to confirm the cauterization point. ESD was performed along the marked points, initially cutting through the mucosal layer and progressively dissecting the submucosal and muscle layers. During the dissection, blue surgical sutures were fortunately identified, confirming the location of the pancreatic-gastric anastomosis ( Fig. 2 ). A small incision was made adjacent to the sutures, allowing the pancreatic fluid to flow out ( Fig. 3 ). Since the pancreatic-gastric anastomosis was observed, the endoscopic retrograde cholangiao-pancreatography (ERCP) technique was applied to cannulate the body part of the pancreatic duct with a guide wire ( Fig. 4 ). The anastomosis was subsequently dilated with a dilating probe. Finally, a pancreatic duct stent was placed using a duodenoscope in combination with ERCP techniques ( Fig. 5 ). Pancreatic fluid drained smoothly into the stomach cavity, and the patient recovered well postoperatively.

**Fig. 1:**
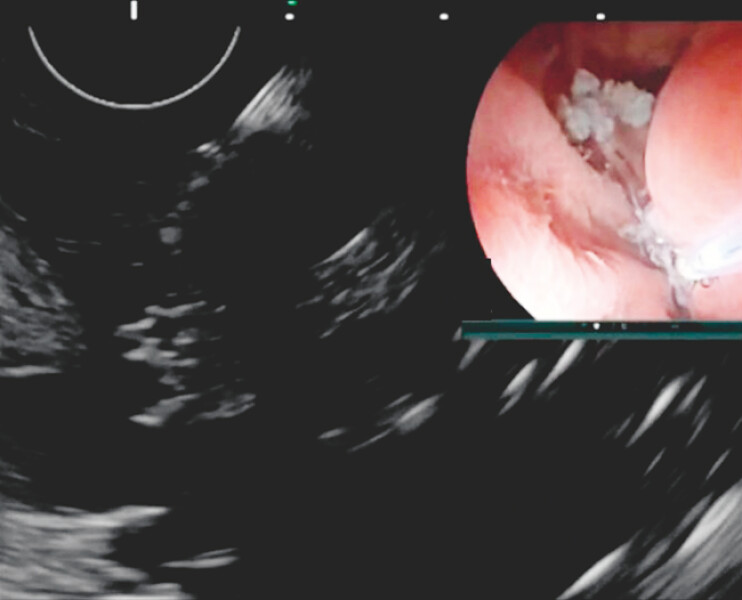
EUS was used to locate the pancreatic duct. The cauterization marking was performed under EUS guidance. Abbreviation: EUS, endoscopic ultrasound.

**Fig. 2:**
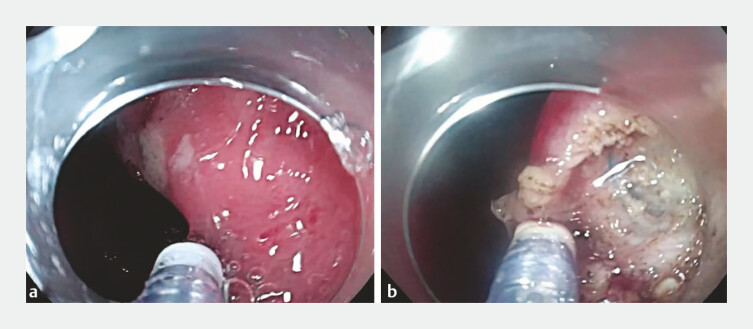
A gastroscope was used to confirm the cauterization point. ESD was performed along the marked points, initially cutting through the mucosal layer and progressively dissecting the submucosal and muscle layer. Abbreviation: ESD, endoscopic submucosal dissection.

**Fig. 3:**
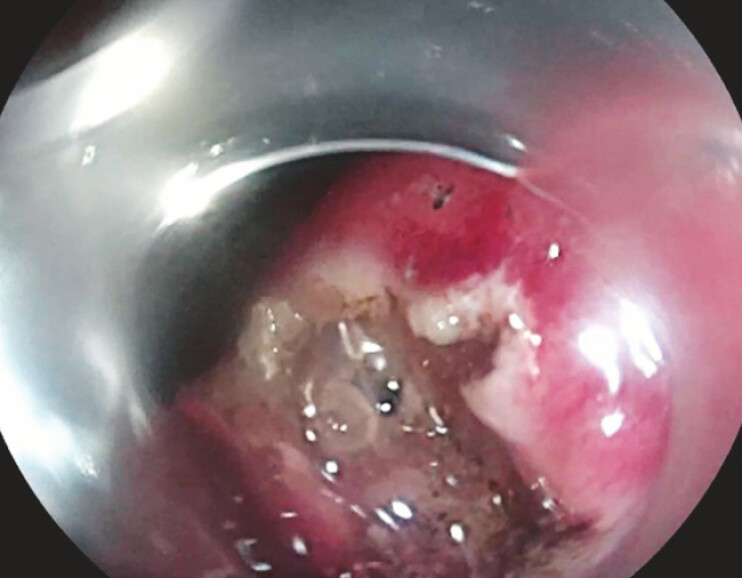
Pancreatic juice flowed into the stomach cavity.

**Fig. 4:**
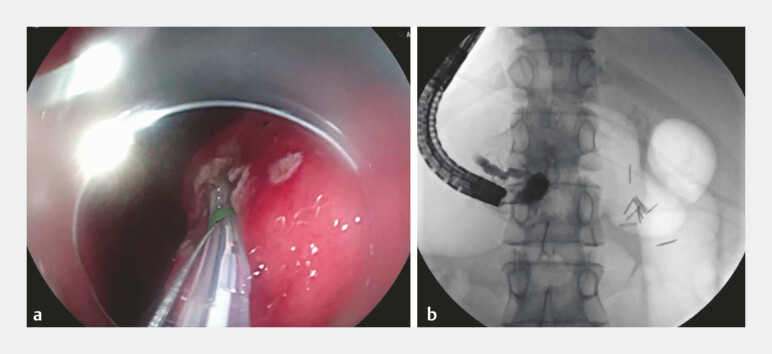
The ERCP technique was applied to cannulate the pancreatic duct with a guide wire. Abbreviation: ERCP, endoscopic retrograde cholangiao-pancreatography.

**Fig. 5:**
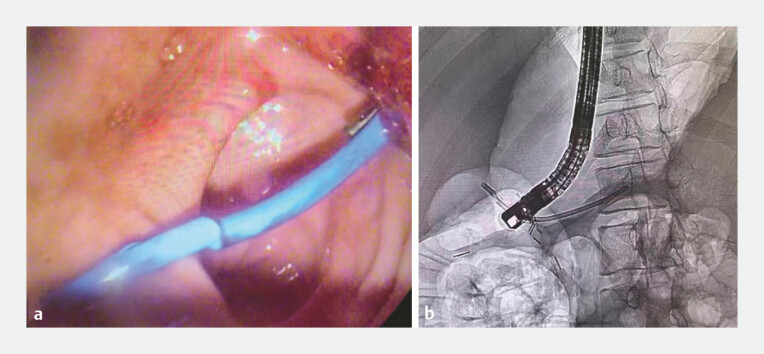
The pancreatic duct stent was placed successfully.

In this case, the precise positioning of EUS, the channel creation of ESD, and the stent placement of ERCP are not merely a combination of techniques but rather a collaborative effort. Through careful planning and a seamless sequence of actions, they successfully resolved the issue of anastomotic stenosis following pancreaticogastrostomy, providing an ideal treatment approach for such diseases.

Endoscopy_UCTN_Code_TTT_1AO_2AD

## Notes

Endoscopy E-Videos https://eref.thieme.de/e-videos
*E-Videos* is an open access online section of the journal *Endoscopy* , reporting on interesting cases and new techniques in gastroenterological endoscopy. All papers include a high-quality video and are published with a Creative Commons CC-BY license. Endoscopy E-Videos qualify for HINARI discounts and waivers and eligibility is automatically checked during the submission process. We grant 100% waivers to articles whose corresponding authors are based in Group A countries and 50% waivers to those who are based in Group B countries as classified by Research4Life (see: https://www.research4life.org/access/eligibility/ ). This section has its own submission website at https://mc.manuscriptcentral.com/e-videos .
